# Supplementary material for: Identification of photoperiod-induced specific miRNAs in the adrenal glands of Sunite sheep (Ovis aries)
Source: Front Vet Sci. 2022 Jul 22;9:888207. doi: 10.3389/fvets.2022.888207 (PMC9354845; doi:10.3389/fvets.2022.888207)
Supplement: Supplementary file 1 [file Data_Sheet_1.ZIP › Supplementary file/miRDeep2 file.zip!/miRDeep2 file/Novel_Structure/LP42A3/Novel_54.pdf]

Provisional ID	:	NC_019475.2_19039
Score total	:	2
Score for star read(s)	:	-1.3
Score for read counts	:	0
Score for mfe	:	1.7
Score for randfold	:	1.6
Score for cons. seed	:	
Total read count	:	21
Mature read count	:	21
Loop read count	:	0
Star read count	:	0

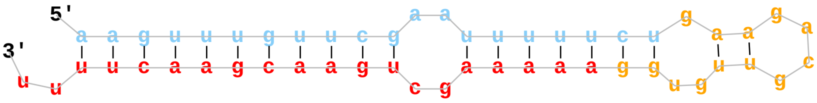

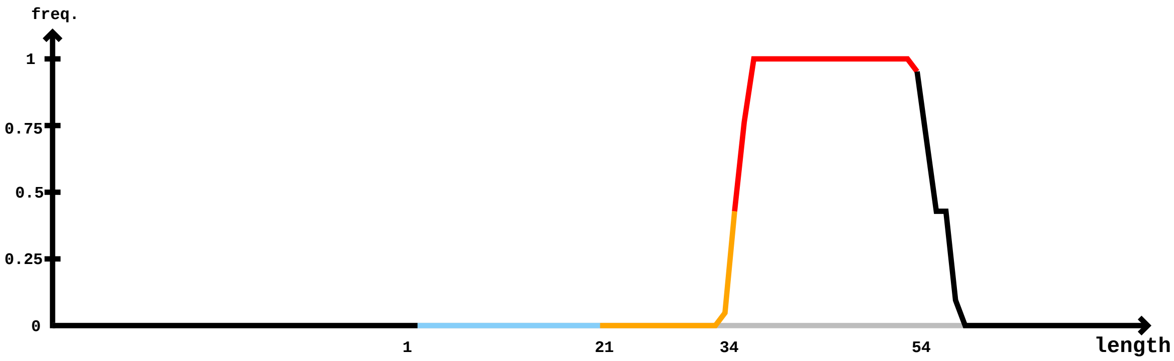

Star Mature

```
5'- gauuugaaaagacauuaugauauugaguuggccgaaaaguuuguucgaauuuuucugaagacguugugg aaaaagcugaacgaacuuuuuggccaaaccaauacaucugccu   -3'    exp
    ................(((.(((((..((((((((((((((((((((..(((((((.((....))..)))))))..))))))))))))))))))))..))))))))......    reads    mm    sample
    ....................................................................gaaaaaCcugaacgaacuuuu.......................    1    1    seq
    .....................................................................aaaaaCcugaacgaacuuu........................    1    1    seq
    .....................................................................aaaaaCcugaacgaacuuuu.......................    7    1    seq
    ......................................................................aaaaUcugaacgaacuuuu.......................    3    1    seq
    ......................................................................aaaaCcugaacgaacuuuuugg....................    1    1    seq
    ......................................................................aaaagcugaacgaacuuuuugg....................    3    0    seq
    .......................................................................aaagcugaacgaacuuuuugg....................    3    0    seq
    .......................................................................aaagcugaacgaacuuuuuggc...................    2    0    seq
```
